# A time-varying SIRD model for dynamic vaccination strategies against COVID-19

**DOI:** 10.1093/eurpub/ckac131.073

**Published:** 2022-10-25

**Authors:** A Cartocci, V Lucarelli, G Messina, N Nante, G Cevenini, P Barbini

**Affiliations:** 1 Department of Medical Biotechnology, University of Siena, Siena, Italy; 2 Department of Molecular and Developmental Medicine, University of Siena, Siena, Italy; 3 Department of Information Engineering and Mathematics, University of Siena, Siena, Italy

## Abstract

**Introduction:**

The COVID-19 pandemic has demonstrated how the optimal allocation of the limited doses of vaccine available represents one of the main useful measures to mitigate the transmission of the infection and reduce the mortality associated with it, especially at an early stage of the pandemic. The use of a compartmental model allows us to understand which population groups to vaccinate and to what extent to act depending on the type of health or social objective to be achieved.

**Methods:**

A time-varying susceptible-infected-recovered-deceased (SIRD) compartmental model, stratified into ten age groups, was developed on Italian data. Simulations were performed every 15 days from December 2020 to April 2021. An optimal vaccination strategy was achieved by minimizing deaths or infected, considering the total vaccine doses available.

**Results:**

We showed how the effects of a vaccination campaign can be planned in a way that maximizes lives saved and/or minimizes infections. Regarding the minimization of deaths, the model prioritizes the elderly (>80 years) and then those between 60 and 80 years, in all simulations. Regarding the cost function of new infections, the first simulation assigns all available doses to those over 90 years of age. In the later simulations, the doses are assigned mainly to the 20-29-year-old and the 89+ year old.

**Conclusions:**

Optimal allocation of available vaccine doses is useful in mitigating transmission of infection and reducing mortality. Application of the mathematical model can be useful at the beginning of an epidemic caused by a new pathogen, when data are scarce, and it is therefore necessary to introduce a standardized approach. This kind of simulation is useful to understand whether the implemented vaccination strategy needs to be recalibrated, too.

**Key messages:**

• Time-varying compartmentalised models can be used both to continuously inform decision-makers about changes in epidemic traits and to simulate the effects of targeted pandemic containment strategies.

• The application of compartmental models can be very useful at the onset of an epidemic to more successfully contain it and structure the health, political, and economic plan.

